# A case series on recurrent and persisting IgA vasculitis (Henoch Schonlein purpura) in children

**DOI:** 10.1186/s12969-023-00872-1

**Published:** 2023-08-14

**Authors:** Julien Marro, Chloe Williams, Clare E. Pain, Louise Oni

**Affiliations:** 1 Department of Women’s and Children’s Health, Institute of Life Course and Medical Sciences, University of Liverpool, Liverpool, UK; 2 Royal Liverpool and Broadgreeen University Hospital Trusts, Liverpool, UK; 3 Department of Paediatric Rheumatology, Alder Hey Children’s NHS Foundation Trust Hospital, Liverpool, UK; 4 Department of Paediatric Nephrology, University of Liverpool Alder Hey Children’s NHS Foundation Trust Hospital, Institute in the Park Building, Eaton Road, Liverpool, L12 2AP UK

**Keywords:** Henoch schonlein purpura, IgAV, Children, Chronic, Recurrent, Persisting, Atypical

## Abstract

**Background:**

IgA vasculitis (IgAV) is a small vessel vasculitis that is more common in childhood. Very limited evidence exists on patients who experience an atypical disease course. The aim of this study was to describe a cohort of children diagnosed with recurrent or persisting IgAV to identify any themes associated with their disease course and areas of unmet needs.

**Methods:**

A single centre retrospective study of children diagnosed with recurrent or persisting IgAV at Alder Hey Children’s Hospital (Liverpool, UK). Clinical data, including features at presentation and during follow up, potential triggers, abnormal laboratory and histology results, treatment and outcome at last clinical review were retrospectively collected. Key themes were identified.

**Results:**

A total of 13 children met the inclusion criteria (recurrent disease, n = 4; persisting disease, n = 9). Median age at first presentation was 10.2 years [2.6–15.5], female:male ratio 1.2:1. Children in the atypical cohort were significantly older than a larger cohort of children who followed a non-complicated disease course (median age 5.5 years (range [0.6–16.7], *p* = 0.003)). All children re-presented with a purpuric rash (either recurring or persisting), accompanied by joint involvement in 92% of patients (12/13). Disease-modifying anti-rheumatic drugs (DMARDs) were used in 8/13 (62%) children. The median time from first presentation to diagnosis of atypical disease was 18.4 months [5.3-150.8] and the time from first presentation to treatment was 24.1 months [1.8–95.4]. Use of corticosteroids was significantly higher in children with renal involvement (*p* = 0.026). During follow up, 8/13 (62%) children were admitted at least once, whilst 10/13 (77%) had re-presented at least once to the emergency department. Five (38%) children were referred to psychology services and 7 (54%) children reported feelings of frustration.

**Conclusions:**

This series describes some characteristics of a small cohort of children with atypical IgAV. It also identifies unmet needs in children with atypical IgAV, which includes delays in diagnosis and lengthy waits for treatment, lack of high-quality evidence regarding treatment choices and a high unrecognised disease burden. Further research is needed to study this subgroup of children as evidence is lacking.

**Supplementary Information:**

The online version contains supplementary material available at 10.1186/s12969-023-00872-1.

## Background

IgA vasculitis (IgAV, formerly Henoch Schonlein purpura, HSP) is the most common vasculitis encountered in childhood with an estimated incidence of 27.2 per 100,000 children in the U.K [1]. It usually presents as a purpuric non-blanching rash most commonly on the lower limbs, although it may extend to the upper limbs and the trunk, and more rarely to the face [2]. During the acute phase, it is accompanied by musculoskeletal involvement in about 80% of patients, in the form of arthralgia and/or oligoarthritis, and gastrointestinal (GI) involvement in up to 75% of patients, which usually presents as colicky abdominal pain that can sometimes precede the rash [2]. Renal involvement (nephritis, IgAVN) is also common in up to 50% of patients with varying degrees of severity [2]. IgAV is a small vessel vasculitis whose pathophysiology remains largely unknown. It is thought to be due to galactose deficient IgA1 immune deposits, although their involvement in non-renal manifestations of the disease remains unclear [3].

IgAV carries an excellent prognosis in most children, with 94% achieving full spontaneous recovery within 2 years [4]. Symptoms typically self-resolve within the first 4 weeks [5] and the main contributor to long term morbidity is renal involvement, with 1–2% estimated to progress onto chronic kidney disease stage 5 (CKD 5) [2]. Another unrecognised medium-to-long term complication is related to relapses: it is estimated that a third of children will experience a relapse [6–8]. Recurrence rates vary in the literature, reported from 2.6 to 66% [7, 9–14], although no formal definition exists perhaps explaining the variation. Several risks factors, such as older age at onset or a more severe form at presentation, have been suggested to predispose patients to relapsing IgAV, but reports are inconsistent [7, 13–16]. Recurrences are described as involving the reappearance of the skin lesions and tend to mimic the first episode in terms of the accompanying organs involved. It usually occurs in the first 6 months following the acute phase, but subsequent episodes are usually milder and shorter than the first episode [7, 8, 11, 14, 16]. Late recurrent episodes, months or even years after the initial presentation, are rare but they are reported [7, 14]. The incidence of renal involvement with recurrent IgAV was reported to be 2.7–11 times higher than in patients without relapsing disease in a meta-analysis [17]. However, the association between recurrent disease and poor outcomes remains unclear [18]. Persisting, unremitting IgAV in children has been reported in several cohort studies but remains poorly defined and is not well documented in the literature [8, 12, 19–22]. Previously, no standardised definitions existed for atypical disease, inspiring the production of national consensus-agreed definitions in the U.K. chaired by the author LO [23].

The aim of this study was to describe a cohort of children diagnosed with atypical (recurrent or persisting) IgAV to identify factors associated with the disease course and areas of unmet needs.

## Methods

### Patient cohort

This was a single-centre retrospective cohort study. Patients were selected to contribute to the cohort if they were children (< 18 years of age at time of disease presentation) who attended Alder Hey Children’s NHS Foundation Trust, Liverpool, UK, with a diagnosis of IgAV. Patients attending between 1st January 2015 to 31st December 2019 were identified by the information technology (IT) team using the International Classification of Diseases (ICD-10) coding system and subsequent patients were identified using those recruited to the IgA Vasculitis Study (established August 2019). Exclusion criteria were as follows: [1] ≥ 18 years old at presentation; [2] diagnosis of IgAV uncertain or in doubt; [3] patients with insufficient available data. From this cohort, patients with a history of recurrent or persisting IgAV were included.

### Definitions

IgAV was diagnosed according to the EULAR/PRINTO/PReS 2008 Ankara endorsed criteria [24]. Due to the lack of standardised definitions, recurrent disease, persisting disease and remission were defined if they had been written in a clinical letter by a paediatric rheumatologist or nephrologist. We describe ‘atypical disease’ as children with either recurrent or persisting disease. Renal involvement was defined as histologically proven IgAV nephritis. From the clinical letters, the main reasons prompting referral to a subspecialist were identified as the main presenting complaint(s), and further symptoms were classified as secondary. Severe cutaneous involvement was defined as a vasculitic rash that was also accompanied by necrotic, bullous and/or ulcerative lesions. The age-specific laboratory reference ranges applied at Alder Hey Children’s NHS Foundation Trust were used to identify out of range blood tests [25, 26].

### Data collection

Clinical data were retrospectively collected from the case notes. Follow up was from diagnosis of IgAV until either discharged or the time of data collection (21st November 2021), whichever occurred first. The history, clinical features, treatment received, and disease progression were recorded at presentation and at each clinical encounter for which data were available. Any triggers that may have proceeded the relapses were recorded from the clinical records if documented, including any evidence of previous streptococcal infections (in the form of ASO titres and/or sputum cultures). In addition, the lowest and highest values of serum C3, C4, IgA, erythrocyte sedimentation rate (ESR) alongside any antinuclear antibodies (ANA) or antineutrophil cytoplasmic antibodies (ANCA) positivity taken at any time during the follow up were recorded. Any histology reports were recorded. The details regarding initial presentation and disease burden were evaluated subjectively as documented by the clinicians. Patients with no prescribed drugs on the electronic system with notes referring to “simple analgesia” or “occasional analgesia” were considered to be taking paracetamol solely. A rash described as “typical of IgAV” at presentation was considered to be a non-severe rash with a typical lower-limb predominant distribution.

### Ethical approval

This study was not considered research by the National Health Service (NHS) Health Research Authority (HRA) as it involved anonymous retrospective data collection. However, some patients were identified through the IgA Vasculitis Study, a single-centre prospective observational study established to develop a clinical cohort of children with IgAV and collect corresponding biological samples to improve the understanding of IgAV in children. The IgAV study consented patients and is conducted at Alder Hey Children’s Hospital (Liverpool, UK). It was fully approved by HRA and Health and Care Research Wales (HCRW) on June 21, 2019 (REC 17/NE/0390, protocol UoL001347, IRAS 236,599).

### Statistical analysis

Clinical and demographic data were compared using Statistical Package for the Social Science (SPSS) version 27.0 software for Windows (IBM Corp, Armonk, NY, USA). Data were assumed to be non-normally distributed due to the small sample size and the Mann Whitney U test was used for continuous variables. The Pearson’s chi-square was applied to categorical variables. Groups were compared as: larger typical IgAV cohort versus atypical cohort, recurrent versus persisting cohorts, renal involvement versus non-renal involvement cohorts. A *p*-value of < 0.05 was considered statistically significant.

## Results

### Patient cohort demographic data and clinical presentation

Between 1st January 2015, and 31st December 2019, 196 children were coded as having a diagnosis of IgAV, of which 42 were excluded (29 were coded incorrectly and 13 had insufficient data). From this cohort, 9 (9/154; 6%) children met the inclusion criteria and had a diagnosis of atypical disease. A further 4 eligible patients were identified from participants of the IgAV study. A total of 13 children were included in this case series. The recurrent and/or persisting cohort were 46% male with a median age at presentation of 10.2 years old (range [2.6–15.5]). Compared to the children with typical disease, 54% of all children with IgAV (n = 145) were male and they were significantly younger with a median age was 5.5 years (range [0.6–16.7], *p* = 0.003).

The characteristics of the atypical cohort are presented in Table 1. From these, 13 children, 4 (4/13; 31%) were diagnosed as having persisting disease while 9 (9/13; 69%) had recurrent IgAV. All children initially presented with a rash, which was accompanied by joint involvement in 8 (8/13; 62%) cases and gastrointestinal (GI) involvement in 7 (7/13; 54%) children. The rash was distributed predominantly on the lower limbs in all patients and extended to the upper limbs, trunk, and face in 5, 2, and 1 child(ren) respectively. Severe cutaneous involvement, in the form of necrotic and/or ulcerative lesions, was present in 2 (2/13; 15%) patients, including in the one with a rash extending to the face. The ankles were the most common joints involved in 4/9 (44%) of the children who initially presented with joint involvement. None of the patients’ initial presenting complaint related to renal involvement. Further clinical data on each individual patient’s presentation is available in Supplementary Table S1.

**Table 1:** Clinical characteristics of children diagnosed with recurrent or persisting IgAV.

	Persisting	Recurrent	Overall	*p*-value
n (%)	4 (31%)	9 (69%)	13 (100%)	-
Male/Female	1/3	5/4	6/7	0.308
Age at diagnosis, years^a^	14.1 [7.9–15.5]	9.1 [2.6–15.1]	10.2 [2.6–15.5]	0.106
**Clinical features at first presentation**				
Rash ^b^	4 (100%)	9 (100%)	13 (100%)	1.000
*Distribution*				
Lower limbs	4 (100%)	9 (100%)	13 (100%)	1.000
Upper limbs	3 (75%)	2 (22%)	5 (38%)	0.071
Trunk	0 (0%)	2 (22%)	2 (15%)	0.305
Face	0 (0%)	1 (11%)	1 (8%)	0.488
Severe cutaneous involvement	1 (25%)	1 (25%)	2 (15%)	0.522
Gastrointestinal involvement ^b^	2 (50%)	5 (56%)	7 (54%)	0.853
Joint involvement ^b^	3 (75%)	5 (56%)	8 (62%)	0.506
Renal involvement^b^	0 (0%)	0 (0%)	0 (0%)	1.000
**Primary reason(s) for referral / re-presentation**				
Rash ^b^	3 (75%)	2 (22%)	5 (38%)	0.071
Gastrointestinal involvement ^b^	1 (25%)	4 (44%)	5 (38%)	0.506
Joint involvement ^b^	3 (75%)	6 (67%)	9 (69%)	0.764
Renal involvement ^b^	3 (75%)	1 (11%)	4 (31%)	**0.021**
Persisting proteinuria/haematuria without renal involvement ^b,c^	0 (0%)	3 (33%)	3 (23%)	0.188
**Symptoms attributable to IgAV at any point during follow up**				
Rash ^b^	4 (100%)	9 (100%)	13 (100%)	1.00
Gastrointestinal involvement ^b^	2 (50%)	4 (44%)	6 (47%)	0.853
Joint involvement ^b^	4 (100%)	8 (89%)	12 (92%)	0.488
Renal involvement ^b^	3 (75%)	1 (11%)	4 (31%)	**0.021**
Persisting proteinuria/haematuria without renal involvement ^b,c^	0 (0%)	4 (44%)	4 (31%)	0.109
Time in months between first diagnosis and diagnosis of recurrent/persisting IgAV^a^	21.0 [5.3–29.4]	13.6 [6.9-150.8]	18.4 [5.3-150.8]	0.940

^a^n (%); ^b^median ; ^c^renal involvement was defined as a urinary albumin to creatinine ratio > 30 mg/mmol. Significant p-value are highlighted in bold text. Due to rounding, percentages may not add up to 100

The main reason prompting referral or re-presentation was joint involvement, in the form of arthralgia, arthritis or both, in 9 (9/13; 69%) children. The median time between the first diagnosis of IgAV and a diagnosis of recurrent and/or persisting IgAV was 18.4 months (range [5.3-150.8]) and this was not significantly different between the two groups. A total of seven (7/13; 54%) patients underwent a skin biopsy to confirm the diagnosis, four (4/13; 31%) had a kidney biopsy, and two (2/13; 15%) had a GI biopsy. All skin biopsies were consistent with a diagnosis of IgAV, however in one case IgA staining was negative but reported as consistent with leukocytoclastic vasculitis in keeping with IgAV. One patient had a repeated skin biopsy due to persisting disease despite initial strong IgA staining and changes consistent with IgAV. Of the two GI biopsies performed, one was reported as normal and the other showed non-specific changes in the colon with focal neutrophilic leukocytes in the lamina propria, consistent with secondary changes seen in IgAV.

### Potential triggers and laboratory findings

One patient had a history of recurrent tonsillitis, one reported having recurrent mouth ulcers, and another had extensive caries at re-presentation. Exercise was reported to trigger flares in three patients, stress in one, and cold weather in another patient. Upper respiratory tract infections (URTIs) were found to precede the onset of relapses in four patients. A total of 8/13 (62%) of patients had laboratory testing for Streptococcal infection during their disease course; 4 (50%) patients had an anti-streptolysin O (ASO) titre, of which 2 (50%) had a strongly positive result (> 800 units/mL), 1 (25%) had a mildly positive result (> 400 but < 800 units/mL), and 1 (25%) had a weakly positive result (> 200 but < 400 units/mL); 3 (38%) patients had both an ASO titre and sputum culture and all were negative (ASO titre < 200 units/mL); 1 (13%) patient had only a sputum culture which was negative for Streptococcus. During follow up, the ESR of 8 patients was elevated outside of their age-specific normal range, one had slightly low complement C3 (1.08 g/L) and another one had low C4 titres (0.10 g/L). All the other patients had normal complement titres, although there was a tendency towards low C4 levels (mean 0.24 g/L; SD ± 0.07). The serum IgA levels were increased above the reference range in five children (38%), whilst four children (31%) were ANA positive. None of the children in this cohort were ANCA-positive.

### Treatment received

The different treatments received by the patients in this cohort is summarised in Table 2. Ten (10/13; 77%) out of the 13 children received analgesia that included paracetamol in all cases, and non-steroidal anti-inflammatory drugs (NSAIDs; i.e., ibuprofen and diclofenac) in 4 (4/13; 31%) cases. Five (5/13; 38%) children required opioids to manage IgAV-related pain. All children with persisting disease had received corticosteroids (CS), either oral or intravenous (IV), whilst only a third of the recurrent group did. Similarly, all the children in the persisting group received disease modifying anti-rheumatic drugs (DMARDS), which included mycophenolate mofetil (MMF), azathioprine, hydroxychloroquine, dapsone and infliximab. Infliximab and intravenous immunoglobulins (IVIG) were used in a case with severe colitis with abdominal pain, rectal bleeding, and vomiting. The treatment decision for this complex case required multi-disciplinary discussions, including paediatric gastroenterologists, nephrologists, and rheumatologists. The decision was based on the findings from the patient’s GI histology and the specialists’ experience with infliximab in other vasculitides, as well as in inflammatory bowel diseases and Behçet’s colitis. Two patients with recurrent purpura received DMARDs (hydroxychloroquine n = 1, dapsone n = 1) without a preceding course of CS. One patient in the persisting group underwent a tonsillectomy mainly due to recurrent episodes of tonsilitis that may have been related to disease flares. This resulted in a significant disease flare in the post-operative period and failed to significantly improve the disease course. The dose and frequency of the DMARDs used in this cohort are presented in Supplementary Table S2.

**Table 2:** Summary of the treatment received by children diagnosed with recurrent or persisting IgAV.

Treatment	Persisting n = 4	Recurrent n = 9	Overall n = 13	*p*-value
Analgesia^a^	4 (100%)	6 (67%)	10 (77%)	0.109
Paracetamol^a^	4	6	10	
NSAIDs^a^	1	3	4	
Opioids^a^	1	4	5	
Corticosteroids^a^	4 (100%)	3 (33%)	7 (54%)	**0.026**
Oral ^b^	Prednisolone - oral (4)	Prednisolone - oral (3)	Prednisolone – oral (7)	
IV ^b^	IV pulsed methylprednisolone (1)	IV pulsed methylprednisolone (1)	IV pulsed methylprednisolone (2)	
DMARDs^a^	4 (100%)	4 (44%)	8 (62%)	0.057
Mycophenolate Mofetil	3	-	3	
Azathioprine	-	3	3	
Hydroxychloroquine	-	2	2	
Dapsone	-	1	1	
Infliximab	1	-	1	
Others				
Management of GI involvement / gastro-protection due to CS use ^a,b^	1 (25%)	4 (44%)	5 (38%)	0.506
Management of proteinuria (ACEi) ^a,b^	2 (50%)	1 (11%)	3 (23%)	0.125
Time in months from first presentation to DMARDs initiation^c^	14.8 [1.8–24.5]	39.0 [23.5–95.4]	24.1 [1.8–95.4]	0.057
Time in months from referral to DMARDs initiation^c^	4.3 [1.2–6.1]	24.3 [13.9–41.4]	10.0 [1.2–41.4]	**0.029**

^a^n (%); ^b^drug name (n); ^c^median. Significant p-value are highlighted in bold text. NSAIDs: non-steroidal anti-inflammatory drugs. IV: intravenous. DMARDs: disease modifying anti-rheumatic drugs. GI: gastrointestinal. ACEi: angiotensin-converting enzyme inhibitor

The median time from first presentation of disease to DMARDs initiation was 14.8 [1.8–24.5] months (recurrent − 39.0 [23.5–95.4]; persisting − 24.1 [1.8–95.4]) whilst median time from specialist referral to DMARDs initiation was 4.3 [1.2–6.1] months. The time from referral to initiation of the DMARDs was significantly lower in patients with persisting disease (median 4.3 months; range [1.2–6.1]) compared to the recurrent group, which was more than 2 years post-referral (median 24.3 months; range [13.9–41.4]; *p* = 0.029).

### Comparison between patients with and without renal involvement

Patients were then grouped according to the presence of renal involvement, to compare the treatment regimens between the subgroups (Table 3). Nine (9/13; 69%) of the atypical patients had non-renal manifestations whereas 4 (4/13; 31%) patients had IgAVN, all of whom had a biopsy consistent with IgAVN. Three were scored using the International Study of Kidney Disease Classification for IgAVN (ISKDC IIIa n = 1; IIIb n = 1; IV n = 1). One patient evolved from persisting IgAV to episodes more consistent with IgA nephropathy and they were scored using the MEST-C score (M-1; E-1; S-0; T-0; C-0). All four patients experienced instances of severely increased albuminuria (maximum urinary albumin-creatine ratio values ranging from 152 to 810 mg/mmol) associated with intermittent episodes of haematuria (all showing 3 + on urine dipstick). Renal function (i.e., serum creatinine values and eGFR) remained normal throughout follow up for all patients with kidney involvement. All patients with renal involvement (4/4; 100%) were treated with CS compared to a third (3/9; 33%) of the patients without (*p* = 0.026). MMF was used in three of the patients with IgAVN, with infliximab in one case, due to a predominance of GI involvement. Hydroxychloroquine, azathioprine and dapsone were used to treat the recurring or persisting non-renal manifestations of IgAV. Although not statistically significant in this small subgroup, there was a trend towards children with IgAVN being treated more promptly with DMARDs (median waiting time from referral to DMARDs initiation 2.9 months [1.2–6.1]) than children without renal involvement (16.6 months [5.6–41.1]; *p* = 0.071).

**Table 3:** Comparison of treatment regimen between patients with and without renal involvement

	Renal involvement (n = 4)	No renal involvement (n = 9)	*p*-value
**Diagnosis**			
Recurrent IgAV/Persisting IgAV^a^	1 (25%) / 3 (75%)	8 (89%) / 1 (11%)	**0.021**
**Corticosteroids**	4 (100%)	3 (33%)	**0.026**
**DMARDs - total** ^a^	3 (75%)	5 (56%)	0.506
Mycophenolate mofetil^a^	3 (75%)	0 (0%)	-
Infliximab^a^	1 (25%)	0 (0%)	-
Hydroxychloroquine^a^	0 (0%)	2 (22%)	-
Azathioprine^a^	0 (0%)	3 (33%)	-
Dapsone^a^	0 (0%)	1 (11%)	-
Time in months between first presentation and DMARDs initiation^b^	8.1 [1.8–24.5]	35.3 [21.6–95.4]	0.143
Time in months between referral and DMARDs initiation ^b^	2.9 [1.2–6.1]	16.6 [5.6–41.4]	0.071

^a^n (%) ; ^b^median ; Renal involvement was defined as a urinary albumin to creatinine ratio > 30 mg/mmol [14]. Significant p-value are highlighted in bold text. DMARDs: disease modifying anti-rheumatic drug

### Follow-up, disease burden and outcomes

Median follow up was 57.7 months [14.3-165.7]. In terms of the disease burden, 5 out of the 13 children (38%; 2/4 in persisting group; 3/9 in recurrent group) were referred to psychology services due to ongoing disease-associated psychological burden. School attendance was affected in 6 (6/13; 46%) and 7 (7/13; 54%) reported feeling self-conscious of the rash and/or frustrated by the refractory symptoms. In addition, 8 (8/13, 62%) were admitted to hospital at least once and 10 (10/13; 77%) children had re-presented to the emergency department due to IgAV. Finally, only three children (23%; 1/4 persisting group; 2/9 recurrent group) had been discharged as of 21st November 2021, two others (15%; 1/4 persisting group; 1/9 recurrent group) were in remission but remained under follow up for kidney monitoring whilst 8 (62%; 2/4 persisting group; 6/9 recurrent group) were still followed up due to ongoing disease. Of the latter patients, 3 (38%) had ongoing single organ symptoms, e.g.: rash or kidney involvement, and the other 5 (62%) patients suffered with symptoms involving 2 or more systems (Supplementary Table S1). At the last clinical review, one patient had a flare of abdominal pain and lower limbs rash following an URTI, resulting in a prescription of a short course of oral corticosteroids and consideration of re-starting azathioprine.

## Discussion

Our study describes a case series of children with atypical IgAV in the form of a recurrent or persisting disease course. This cohort demonstrates that despite most children experiencing a self-limiting disease course, IgAV can persist in a minority leading to complex management and a higher disease burden.

We report some of the previously described characteristics of children with an atypical course, which include older age at onset, a less typical rash distribution, and potential triggers. In the current study, children with recurrent/persisting disease were significantly older than a larger cohort of children who followed an uncomplicated disease course, supporting that older age may be a risk factor. Increasing age at onset (usually over 8 years old) has been identified as a risk factor by several studies for relapsing IgAV [12–14, 27] whilst others did not find this association [7, 11, 12, 16, 20, 28]. Liao et al. suggested that the relative importance of white blood cells and IgA levels in the pathogenesis of IgAV may change with age, possibly contributing to the higher frequency of atypical disease reported in older children [12].

In this series, all patients presented with a lower limb predominant rash and 5 (5/13; 38%) also demonstrated an upper limb distribution with trunk involvement in one and with both trunk and facial involvement in another. Additionally, a recent multicentre study reported that patients with a generalised rash, necrotic or ulcerative skin lesions, or a rash extending to the upper limbs had higher rates of relapse compared to children with a purpura confined to the lower limbs [15], however further large studies are required to confirm this association. Chronic tonsillitis and other ENT conditions may be associated with recurrent purpura [29], although only three children from our cohort (23%) were reported to suffer from such problems. Larger cohort studies are required to assess the influence of various ENT pathologies on atypical IgAV. Interestingly, 3 patients (3/13; 23%) described exercise-induced flares of IgAV. This aligns with a recent prospective study of 121 children with IgAV which suggested limiting strenuous exercise helped to reduce the recurrence rate [30]. Streptococcal infection is the most frequent and well-recognised trigger of IgAV in children [31] and our case series identified heterogeneity in the performing testing for Streptococcal infection. ASO titres are probably considered the optimal investigation however they are not completely sensitive [32]. Due to the heterogeneity in the sampling in this cohort it is difficult to confirm whether it was a true trigger prior to the disease or flare onset. The literature suggests that whilst Streptococcal infection is a trigger for IgAV, it is not associated with either relapse or recurrence, or with a worse renal prognosis [31]. We reported ANA positivity in 4 (4/13; 31%) children, consistent with that previously described by Calvo Rio et al. who reported a significantly increased incidence of ANA-positivity in a large cohort of children with relapsing IgAV compared to children without relapses (27.3% vs. 8.3%) [16]. However, up to 15% of all healthy children will have a positive ANA test [33] and there are discrepancies in the literature on whether some laboratory parameters could be more predictive/indicative of atypical disease [7, 16, 34, 35]. Some studies have suggested that renal involvement may predispose to recurrent disease [11] and that persistent purpura was a risk factor for the development of IgAVN [17], although it does not necessarily correlate with poorer outcomes [18]. Our study found that no atypical patients initially presented with kidney involvement however patients with persisting disease had a much higher rate of kidney involvement than those with recurrent disease during follow up. This was also reported by a larger study including 484 children with IgAV in which renal involvement was associated to a higher degree with refractory disease (OR 20.51, 95%CI [10.73–41.84], *p* < 0.001) than recurrent disease (OR 3.05, 95%CI [1.42–6.49], *p* = 0.004) [12]. This may be due to persisting disease involving more systemic and prolonged inflammation, or may be cofounded by the age of onset which is a recognised risk factor for both refractory disease and kidney involvement. Nonetheless, more studies are needed to investigate the complex interplay between atypical disease and kidney involvement.

There are little data in the literature regarding the frequency of relapsing symptoms. We found that all patients re-presented with a persisting rash with concomitant joint involvement in the majority (12/13; 92%) and GI involvement in 6/13 (47%). Several studies have reported the most common re-presenting symptoms were a rash associated with joint involvement (24-67%), often followed by GI involvement (recurring symptoms in 17-67%) [7, 8, 10, 19, 35–38].

In 2019, the first European consensus-based recommendations for diagnosis and treatment of paediatric IgAV were published, however no recommendations were made for children with refractory non-renal manifestations [39]. The use of CS in atypical IgAV is controversial. Patients with severe symptoms may benefit from early CS treatment as previous clinical trials have shown that prednisone may reduce extrarenal symptoms and improve clinical outcomes in these cases [14, 40, 41]. However, the efficacy of CS in mild purpura recurrence or persistence remains unknown and prolonged use of CS should be minimised due to the high frequency of side effects which are unfavourable for paediatric patients [42]. Evidence supporting the efficacy of azathioprine and MMF is based on small case series and case reports [43, 44], and there is no convincing evidence to support the use of hydroxychloroquine and dapsone [45–47], despite it being used in the current series mainly for skin manifestations. These DMARDs are currently being investigated in a clinical trial recruiting adults with cutaneous manifestations of IgAV (ClinicalTrials.gov; NCT02939573; colchicine vs. dapsone vs. azathioprine). One patient in our cohort received infliximab and IVIG to treat severe colitis. Interestingly, the anti-TNF monoclonal antibody infliximab has been reported to possibly trigger IgAV [3, 48] and there are no other reports of infliximab use for complex IgAV in the literature. IVIG is rarely used, but the few case-reports available in the literature did report successful treatment of severe GI involvement [49–52]. One clinical trial is underway to assess the efficacy of IVIG versus methylprednisolone in children with IgAV and GI involvement refractory to ordinary dosage of prednisolone (ClinicalTrials.gov; NCT03647852). Although not used in our cohort, methotrexate and rituximab may also play a role in treating refractory paediatric IgAV [53–55], with the latter currently being evaluated in a trial recruiting adult patients with relapsing IgAV (ClinicalTrials.gov; NCT05329090).

Whilst this small series highlights the range of treatment choices for children with atypical disease, it also highlights the delay in starting treatment for many patients, with those suffering from renal manifestations being treated more promptly. IgAV can have a significant psychological impact which is likely to be underestimated, especially in adolescents where systemic manifestations can affect self-image and participation in activities. As illustrated in this series, diagnosing atypical disease can take a long time, with long waits for initiation of specialist treatment and the need for further investigation which may add frustration for patients and families. A recent study found that parents’ ability to cope with stress was negatively correlated with the frequency of their child’s IgAVN recurrence [56]. A holistic multidisciplinary approach is therefore needed for managing patients with atypical IgAV.

The lack of standardised definitions for relapsing and persisting disease likely hinders the accuracy and applicability of the current literature and impacts on clinical delays. The recent UK consensus-agreed guidelines for IgAV management in children has proposed definitions of atypical disease with persisting disease being defined as patients with a typical rash lasting more than one month, while recurrent IgAV was defined as a reappearance of the typical rash after a symptom-free period of greater than 1 month [23]. International agreement on standardised definitions is needed to provide a catalyse for more robust studies.

This study has several limitations. Firstly, those associated with retrospective data collection which included limited baseline data on patients who did not initially present to our centre. Additionally, assumptions were made regarding rash distribution for such patients and simple analgesia was presumed to be paracetamol where not stated. A major limitation was the lack of standardised definition for recurrent and/or persistent disease and we had to rely on the clinician’s diagnosis. We are therefore likely to have under reported the true incidence of atypical disease in children with IgAV.

## Conclusion

The current case-series has identified areas of unmet needs in children with recurrent or persisting IgAV. This includes a long wait from presentation to diagnosis and treatment, lack of evidence regarding treatment choices in this population, and a high disease-burden. Further research and high-quality clinical trials are urgently needed to further assess children with an atypical IgAV disease course.

### Electronic supplementary material

Below is the link to the electronic supplementary material.

Supplementary Material 1

## Data Availability

The datasets used and/or analysed during the current study are available from the corresponding author on reasonable request.

## List of abbreviations

ACEi: Angiotensin-converting enzyme inhibitor
ANA: Anti-nuclear antibody
ANCA: Antineutrophil cytoplasmic antibodies
CKD: Chronic kidney disease
CS: Corticosteroids
DMARDs: Disease-modifying anti-rheumatic drugs
ENT: Ear, nose and throat
ESR: Erythrocyte sedimentation rate
HRA: Health Research Authority
ICD-10: International Classification of Disease 10th Revision
IgAV: IgA vasculitis
IgAVN: IgA vasculitis with nephritis
ISKDC: International Study of Kidney Disease in Children
IV: Intravenous
IVIG: Intravenous immunoglobulins
MMF: Mycophenolate mofetil
NHS: National Health Service
URTI: Upper respiratory tract infection
