# Supplementary material for: A case series on recurrent and persisting IgA vasculitis (Henoch Schonlein purpura) in children
Source: Pediatr Rheumatol Online J. 2023 Aug 14;21:85. doi: 10.1186/s12969-023-00872-1 (PMC10424434; doi:10.1186/s12969-023-00872-1)
Supplement: Supplementary file 1 — Supplementary Material 1 [file 12969_2023_872_MOESM1_ESM.docx]

**A case series on recurrent and persisting IgA vasculitis (Henoch Schonlein purpura) in children**

*Julien Marro^1^, Chloe Williams^2^, Clare E. Pain ^1,3^, Louise Oni^1,4^*

*^1^Department of Women’s and Children’s Health, Institute of Life Course and Medical Sciences, University of Liverpool, Liverpool, United Kingdom*

*^2^ Royal Liverpool and Broadgreeen University Hospital Trusts, Liverpool, United Kingdom*

*^3^Department of Paediatric Rheumatology, Alder Hey Children’s NHS Foundation Trust Hospital, Liverpool, United Kingdom*

*^4^Department of Paediatric Nephrology, Alder Hey Children’s NHS Foundation Trust Hospital, Liverpool, United Kingdom*

# Supplementary tables

[Table S 1: Summary of the demographics and clinical characteristics of individual patients in the case series. 2](#_Toc140243946)

[Table S 2: Summary of DMARDs treatments used in individual patients of the series. 4](#_Toc140243947)

Table S 1: Summary of the demographics and clinical characteristics of individual patients in the case series.

|  | **Sex** | **Age at diagnosis (years)** | **Recurrent or persistent disease** | **Symptoms at initial presentation** | | | | **Symptoms prompting re-presentation** | **Outcome** | **Reason for on-going follow up** | **Treatment at follow-up** |
| --- | --- | --- | --- | --- | --- | --- | --- | --- | --- | --- | --- |
|  |  |  |  | **Cutaneous involvement** | **Joint involvement** | **Gastrointestinal involvement** | **Kidney involvement** |  |  |  |  |
| **Patient 1** | M | 15.1 | Recurrent | LL, UL, trunk | Swollen legs, hands | No | No | Persistent proteinuria | Discharged | N/A | N/A |
| **Patient 2** | M | 12.8 | Recurrent | LL | No | Abdominal pain | No | Arthralgia | Follow-up | On-going flares of petechial rash | Nil |
| **Patient 3** | F | 9.1 | Recurrent | LL | Fingers, wrists, hip, knees | Abdominal pain | No | Arthralgia | Discharged | N/A | N/A |
| **Patient 4**^a^ | F | 7.9 | Persisting | LL | No | Abdominal pain  PR bleeding  Vomiting | No | Colitis (abdominal pain, PR bleed, vomiting), arthralgia, Kidney involvement | Discharged | N/A | N/A |
| **Patient 5** | M | 6.2 | Recurrent | LL | No | Abdominal pain, vomiting | No | Kidney involvement  Abdominal pain | Follow-up | On-going kidney monitoring, currently in remission | Nil |
| **Patient 6** | M | 2.6 | Recurrent | LL | No | No | No | Persistent proteinuria  Arthralgia | Follow-up | Recurrent rash | Nil |
| **Patient 7^b^** | F | 15.5 | Persisting | LL, UL | Ankles | Abdominal pain | No | Rash  Kidney involvement | Follow-up | Persisting proteinuria | MMF (1000mg BD) |
| **Patient 8** | M | 12.7 | Recurrent | LL | No | Abdominal pain | No | Abdominal pain | Follow-up | Recent flare with on-going abdominal pain and rash | Prednisolone 40mg OD^c^  Omeprazole 20mg OD |
| **Patient 9** | F | 7.2 | Recurrent | LL, UL, trunk, face | Ankles | Vomiting, abdominal pain | No | Abdominal pain  Arthralgia | Follow-up | Recurrent flares with abdominal pain, rash, and arthralgia/arthritis in ankles | AZA (150mg OD) |
| **Patient 10** | F | 3.4 | Recurrent | LL | Ankles, feet | No | No | Rash, abdominal pain, arthralgia, intermittent proteinuria/ macroscopic haematuria | Follow-up | Flares three to four times per year with rash, severe abdominal pain, joint involvement, and haematuria/proteinuria | Nil |
| **Patient 11** | M | 13.8 | Persisting | LL, UL | Joints involved unknown | No | No | Rash, arthralgia | Follow-up | Flares over winter with rash, with ankle and knee arthralgia | AZA (200mg OD) |
| **Patient 12** | F | 14.4 | Persisting | LL, UL | Ankles | No | No | Rash, arthralgia, joint swelling, kidney involvement | Follow-up | On-going kidney monitoring, currently in remission | MMF (750mg BD) and lisinopril (10mg OD) |
| **Patient 13** | F | 10.2 | Recurrent | LL | Joins involved unknown | No | No | Rash. arthralgia, joint swelling | Follow-up | On-going rash, arthralgia, and kidney involvement | Dapsone (100mg OD) |

*LL: lower limbs; UL: upper limbs; AZA: azathioprine; MMF: mycophenolate mofetil. OD: once a day. BD: twice a day. ^a^Patient 7 suffered from severe skin lesions that left marks and ulceration on the right leg. ^b^Patient 9 had some severe necrotic areas with some blistering at times. ^c^At last review this patient had an ongoing IgAV flare resulting in a course of corticosteroid: initially prednisolone 40mg OD for 5 days then 20mg OD for 5 days, then 10mg OD for 5 days then stop. This patient was under consideration to re-start azathioprine.*

Table S 2: Summary of DMARDs treatments used in individual patients of the series.

|  | **Treatment 1** | **Duration** | **Treatment 2** | **Duration** | **DMARD at follow-up** |
| --- | --- | --- | --- | --- | --- |
| **Patient 1** | Nil |  |  |  | N/A –discharged |
| **Patient 2** | Hydroxychloroquine (200mg OD) | 6 months |  |  | Nil |
| **Patient 3** | Nil |  |  |  | N/A – discharged |
| **Patient 4** | MMF (500mg BD), then increased to 750mg OM / 500mg ON | 2 years 2 months | Infliximab 6mg/kg – initially 4 weekly then according to response/symptoms | On/off over 3 years according to symptoms | N/A – discharged |
| **Patient 5** | Nil |  |  |  | Nil |
| **Patient 6** | Nil |  |  |  | Nil |
| **Patient 7** | MMF 250mg BD initially, increased to 1000 mg BD by 250mg BD increments over a 2 years period | 2 years *(continued)* |  |  | MMF (1000mg BD) |
| **Patient 8** | AZA (85mg OD) then increased to 100mg OD | 1 year 3 months |  |  | Nil |
| **Patient 9** | Hydroxychloroquine (100mg OD) | 1 year 3 months | AZA (50mg OD) increased to 100mg OD and then 150mg OD | 1 year *(continued)* | AZA (150mg OD) |
| **Patient 10** | Nil |  |  |  | Nil |
| **Patient 11** | AZA (100mg OD) increased to 150mg OD then 200mg OD | 1 year *(continued)* |  |  | AZA (200mg OD) |
| **Patient 12** | MMF (250mg BD), increased to 500mg BD then 750mg BD | 1 year 8 months *(continued)* |  |  | MMF (750mg BD) |
| **Patient 13** | Dapsone (100mg OD) | 1 month *(continued)* |  |  | Dapsone (100mg OD) |

*AZA: azathioprine; MMF: mycophenolate mofetil. OD: once a day; OM: every morning; ON: every night; BD: twice a day.*
